# The nonlinear variation of drought and its relation to atmospheric circulation in Shandong Province, East China

**DOI:** 10.7717/peerj.1289

**Published:** 2015-10-27

**Authors:** Baofu Li, Zhongsheng Chen, Xingzhong Yuan

**Affiliations:** 1 College of Geography and Tourism, Qufu Normal University, Rizhao, China; 2 The Key Laboratory of Geographic Information Science, Ministry of Education, East China Normal University, Shanghai, China; 3 State Key Laboratory of Coal Mine Disaster Dynamics and Control, Chongqing University, Chongqig, China

**Keywords:** PDSI, Nonlinear trend, PDO, ENSO, Multi-scale, East China

## Abstract

Considerable attention has recently been devoted to the linear trend of drought at the decadal to inter-decadal time scale; however, the nonlinear variation of drought at multi-decadal scales and its relation to atmospheric circulation need to be further studied. The linear and nonlinear variations of the Palmer drought severity index (PDSI) in Shandong from 1900 to 2012 and its relations to the Pacific decadal oscillation (PDO), El Niño-Southern Oscillation (ENSO), Siberian high (SH) and Southern Oscillation (SO) phase changes from multi-scale are detected using linear regression, the Mann–Kendall test, ensemble empirical mode decomposition (EEMD) and the Pearson correlation analysis method. The results indicate that the PDSI shows no statistically significant linear change trend from 1900 to 2012; however, before (after) the late 1950s, PDSI shows a significant upward (downward) trend (*P* < 0.01) with a linear rate of 0.28/decade (−0.48/decade). From 1900 to 2012, the PDSI also exhibits a nonlinear variation trend at the inter-annual scale (quasi-3 and quasi-7-year), inter-decadal scale (quasi-14-year) and multi-decadal scale (quasi-46 and quasi-65-year). The variance contribution rate of components from the inter-annual scale is the largest, reaching 38.7%, and that from the inter-decadal scale and multi-decadal scale are 18.9% and 19.0%, respectively, indicating that the inter-annual change exerts a huge influence on the overall PDSI change. The results also imply that the effect of the four atmospheric circulations (PDO, ENSO, SH, SO) on PDSI at the multi-decadal variability scale are more important than that at the other scales. Consequently, we state that PDSI variation at the inter-annual scale has more instability, while that at the inter-decadal and multi-decadal scale is more strongly influenced by natural factors.

## Introduction

Water is the lifeline of the survival and development of human society. Water issues primarily include two aspects: one is the shortage of water resources, and the other is severe water pollution. As one of the poorest countries in the world per capita water resources, China occupies only a quarter of the entire globe with respect to fresh water resource per capita. Particularly, water resource per capita in the Shandong province is less than 1/6 of the average level of China. Water shortage has become a “bottleneck” for the sustainable development of the social economy in Shandong. Meanwhile, against the background of the global water cycle’s being affected by climate change, the drought in Shandong has seriously hindered the development of the local social economy in recent years. For example, in 2014, the drought in Shandong resulted in economic losses totaling more than 3.9 billion Yuan. Therefore, the urgent need is to extensively study the drought change in Shandong and its influence factors.

The Palmer drought severity index (PDSI) is one of the common methods used to reflect the characteristics of climate system change in an area reasonably (Dai, 2011a; Dai, 2011b). Furthermore, Wei & Ma (2003) found that the PDSI can accurately reflect the drought in eastern China. Meanwhile, many previous studies have focused on the linear change trend of drought in China over the most recent 50 years (Qi, Zhi & Bai, 2011; Zhang et al., 2015a) and have stated that since the late 1990s, most of northern China (except for western Northwest China) has undergone severe and prolonged dry periods (Zou, Zhai & Zhang, 2005; Zhang et al., 2015b). Nevertheless, as a complex nonlinear system, long-term climate system variations, such as drought, display nonlinear, non-stationary complex processes of change at a variety of scales or with periodic oscillations (Xu et al., 2013; Bai et al., 2014; Franzke, 2014). The previous methods could not illustrate the natural variability of climate change accurately and reasonably to some degree. Based on signal detection technology, Wu & Huang (2009) came up with the ensemble empirical mode decomposition (EEMD), a new time series signal processing method. As a new development of empirical mode decomposition (EMD), this method features stronger self-adaptability and local variation characteristics. These attributes improve the “mode mixing” issue of EMD and detect non-stationary and nonlinear processes effectively, separating the oscillations at different scales (intrinsic mode function, IMF) or the trend components from the original signal gradually (Wu & Huang, 2009). EEMD is one of the latest methods to more efficiently extract trends and periodic information (Li, Chen & Shi, 2012). Furthermore, the EEMD method has been widely applied to study climate change (Wu et al., 2011; Kuo, Gan & Chan, 2013; Ji et al., 2014; Qian & Zhou, 2014).

In recent years, previous studies have mainly studied drought from the decadal to inter-decadal time scale in northern China (Ma, 2007; Qi, Zhi & Bai, 2011). There is limited research on drought variability at the multi-decadal scale for the entire twentieth century (Qian & Zhou, 2014). Moreover, the drought variability of dry-wet evolution at the multi-decadal scale, especially the recent drying trend in northern China, remains unclear. Additionally, atmospheric circulation (such as PDO, and ENSO) has been considered to be one of the important factors that affect drought (Ma & Shao, 2006; Ma, 2007; Wang, Chen & Zhang, 2012). The correlation between drought or climate and atmospheric circulation from the decadal to inter-decadal time scale has been detected in many studies (Ma & Shao, 2006; Ma, 2007; Li, Chen & Shi, 2012; Xiao, Zhang & Singh, 2014). However, there are only limited studies investigating the drought or climate relations to atmospheric circulation at multi-decadal scales. Thus, this study aims to explore the following issues: (1) the linear and nonlinear change trend of PDSI for Shandong from 1900 to 2012 at the inter-annual, inter-decadal and multi-decadal scales; (2) the contributions of oscillations at different scales to PDSI changes; and (3) the relationship between PDSI changes and Pacific decadal oscillation (PDO), El Niño-Southern Oscillation (ENSO), Siberian High (SH), and Southern Oscillation (SO) at the inter-annual, inter-decadal and multi-decadal scales.

Many previous studies have implied that a phase transition of the Pacific decadal oscillation enables the East Asian summer monsoon to have a weakening tendency (Ma, 2007; Li et al., 2010). Qian & Zhou (2014) stated that from 1960 to 1990, approximately 70% of the drying trend in northern China stemmed from 50- to 70-year multi-decadal variability, which is related to Pacific decadal oscillation (PDO) phase changes. ENSO is characterized by large-scale sea surface temperature (SST) anomalies in the eastern equatorial Pacific Ocean. The most direct effect of ENSO is an interaction in surface pressure related to a modulation of trade winds and a shift of tropical Pacific precipitation (Bellenger et al., 2014). The Siberian High (SH) is a cold or very cold dry air mass that forms in the Mongolian-Siberian region. It has a huge impact on the weather patterns in mid-to-high latitude Asia (Li, Chen & Shi, 2012). The Southern Oscillation (SO) in the tropical ocean-atmosphere system is the dominant interannual climate phenomenon. Therefore, ENSO, PDO, SH, and SO are likely to have an effect on the climate system for the study area.

## Study Area, Data, and Methods

### Study area

**Figure 1:**
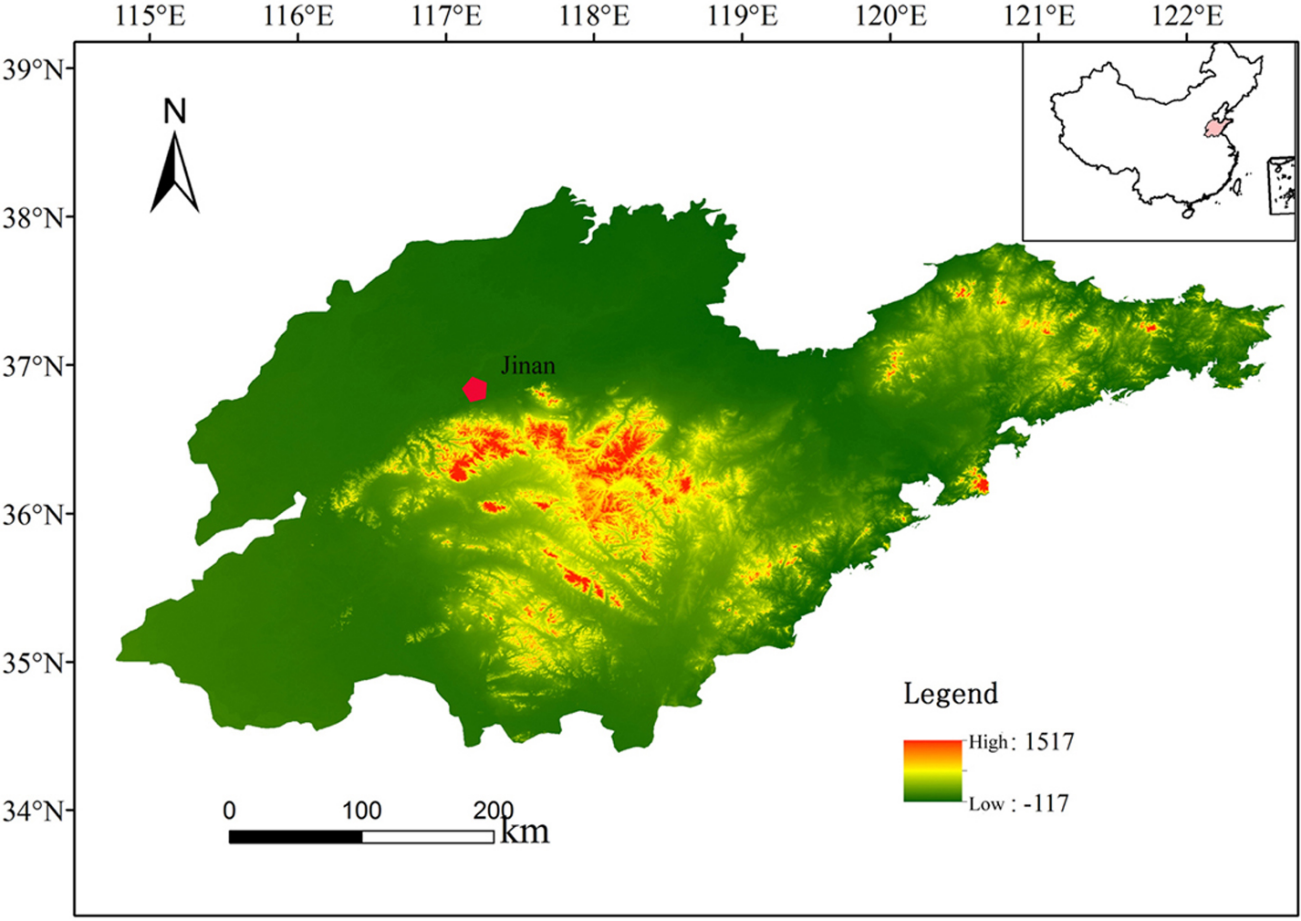
Location map of the study area.

The Shandong province is located in the lower reaches of the Yellow River on China’s east coast between E114°36′–E122°43′ and N34°25′–38°23′ (Fig. 1). This province covers a total area of 15.67 square kilometers. accounting for 1.6% of the nation’s total, and extends approximately 700 km from west to east and 420 km from north to south. Shandong weather features a mild climate with concentrated rainfall, and four clear seasons, which belongs to the continental monsoon climate of the warm temperate zone. Southerly winds prevail in summer, which is hot and rainy, while northerly winds prevail in winter, which is cold and dry; spring weather is changeable, with drought, less rain and much wind and dust, while autumn weather is sunny and cool with a moderate temperature. The annual average temperature in Shandong is 11 °C–14 °C; annual sunshine hours are 2300–2300 h; the annual average rainfall is 550–950 mm, concentrated mostly from June to September; water resources per capita occupy 334 cubic meters, being only 1/6 of the national per capita and 1/25 the global per capita.

### Data

PDSI data are employed for the period 1850–2012, archived at http://www.cgd.ucar.edu/cas/catalog/climind/pdsi.html. The data are available on a 2.5°× 2.5° grid and cover global land area between 60°S and 77.5°N (Dai, 2011a). In this article, the Shandong Province of East China refers to the area generally defined by N35°–N37.5° and E115°–E122.5°. Therefore, this study used the average of the values from the N35°–N37.5° and E115°–E122.5° grids as the representative value of the Shandong PDSI. Based on the PDSI values (Palmer, 1965), drought and wet periods are classified in Table 1.

**Table 1:** Classes for drought and wet periods based on PDSI value.

Value	Classes	Value	Classes
≤−4.00	Extreme drought	1.0∼1.99	Slightly wet
−3.0∼−3.99	Severe drought	2.0∼2.99	Moderately wet
−2.0∼−2.99	Moderate drought	3.0∼3.99	Very wet
−1.0∼−1.99	Mild drought	≥4.00	Extremely wet
0.99 ∼−0.99	Normal		

The PDO index monthly data (1900–2010) used in this paper are from http://research.jisao.washington.edu/pdo/PDO.latest. The ENSO data are from http://research.jisao.washington.edu/data_sets/globalsstenso. The monthly data of SOI for 1900–2010 for this study are from http://www.cgd.ucar.edu/cas/catalog/climind/SOI.signal.annstd.ascii. The authors and Li, Chen & Shi (2012) quote the Siberian High intensity index (SHI) defined by Gong & Wang (1999). We used the monthly mean sea level pressure data of the northern hemisphere provided by the Hadley Center (HadSLP) (Allan & Ansell, 2006) to calculate the SHI for 1900–2010. These atmospheric circulations have a certain impact on the climate of China.

Pearson’s correlation coefficient is used to detect the relationship between the PDSI and each of the atmospheric circulation indices.

### Methods

#### Mann–Kendall statistical test

We used the Mann–Kendall (MK) statistical test (Mann, 1945; Kendall, 1975) to test the significances of trends in the annual mean PDSI of the study area. The nonparametric Mann–Kendall statistical test has been commonly used to assess the significance of monotonic trends in meteorological and hydrologic series. For a time series *X* = □*x*_1_, *x*_2_, …, *x_n_*□, when *n* > 10, the standard normal statistic *Z* is estimated as follows:

(1) \begin{eqnarray*} Z=\left\{\begin{array}{ll} \displaystyle \left(S-1\right)/\sqrt{\mathrm{var}\left(S\right)}&\displaystyle S> 0\\ \displaystyle 0&\displaystyle 0\\ \displaystyle \left(S+1\right)/\sqrt{\mathrm{var}\left(S\right)}&\displaystyle S\lt 0 \end{array}\right. \end{eqnarray*}

where

(2) \begin{eqnarray*} S=\sum _{i=1}^{n-1}\sum _{j=i+1}^{n}\mathrm{sgn}\left({x}_{j}-{x}_{i}\right) \end{eqnarray*}

(3) \begin{eqnarray*} \mathrm{sgn}\left(\theta \right)=\left\{\begin{array}{ll} \displaystyle +1,&\displaystyle \theta > 0\\ \displaystyle 0,&\displaystyle \theta =0\\ \displaystyle -1,&\displaystyle \theta \lt 0 \end{array}\right. \end{eqnarray*}

(4) \begin{eqnarray*} \mathrm{var}\left(S\right)=\left[n\left(n-1\right)\left(2 n+5\right)-\mathop{\sum }\limits _{t}t\left(t-1\right)\left(2 t+5\right)\right]/18 \end{eqnarray*}

where *t* is the extent of any given time and $\sum _{t}$ denotes the summation of all ties.

The magnitude of the trend is given as

(5) \begin{eqnarray*} \beta =\mathrm{Median}\left(\frac{{x}_{i}-{x}_{j}}{i-j}\right),\hspace{1em}{\forall }_{j}\lt i \end{eqnarray*}

in which 1 < *j* < *i* < *n*. A positive value of *β* indicates an ‘upward trend’ and a negative value of *β* indicates a ‘downward trend’.

With the null hypothesis (H0) of *β* = 0, where *β* is the slope of trend, the MK test rejects H0 if |*Z*| > *Z*_1−*α*/2_, in which +*Z*_1−*α*/2_ and −*Z*_1−*α*/2_ are the standard deviates and *α* is the significance level for the test. In this study, we use significance levels of *α* = 0.05, with corresponding standard normal deviates of 1.96.

#### Ensemble empirical mode decomposition

We applied the ensemble empirical mode decomposition (EEMD) method (Wu & Huang, 2009; Huang & Wu, 2008) to decompose the PDSI and atmospheric circulation indices during the period of 1900–2012 (2010) into different time-scales and its nonlinear trend. The EEMD method defines the true IMF components as the mean of an ensemble of trials, each consisting of a signal plus white noise of finite amplitude (Wu & Huang, 2009). This method is a new development of the EMD method (Huang et al., 1998) to overcome the scale-mixing problem. In the EEMD method, a time series *x*(*t*) is decomposed in terms of adaptively obtained, amplitude–frequency modulated oscillatory components *C_i_* (*i* = 1, 2, …, *n*) and a residual *R_n_*, a curve that is either monotonic or contains only one extremum from which no additional oscillatory components can be extracted:

(6) \begin{eqnarray*} x(t)=\sum _{i=1}^{n}{C}_{i}(t)+{R}_{n}(t). \end{eqnarray*}

Compared with many traditional methods specifying a priori basis functions, such as Fourier transform, wavelet analysis, and curvelet transform, EEMD uses natural wave forms, which guarantees that the physical interpretation within specified time intervals does not change with the addition of new data, according to a physical constraint in which the subsequent evolution of a physical system cannot alter the reality that has already occurred (Ji et al., 2014; Bai et al., 2014).

To determine different scales of IMF components, we examined a more detailed distribution of the energy with respect to the period in the form of a spectral function. The energy density of the *i*th IMF components (*E_i_*) can be defined as follows:

(7) \begin{eqnarray*} {E}_{i}=\frac{1}{N}\sum _{j=1}^{N}{\left\vert {I}_{i}(j)\right\vert }^{2} \end{eqnarray*}

where *N* is the length of the IMF component and *I_i_*(*j*) denotes the *i*th IMF component. The white noise sequence is tested using the Monte Carlo method (Wu & Huang, 2004); then, a simple equation that relates the energy density (${\bar {E}}_{i}$) and the averaged period (${\bar {T}}_{i}$) is obtained:

(8) \begin{eqnarray*} \lg ~\bar {{E}_{i}}+\lg {\left(\bar {{T}_{i}}\right)}_{\alpha }=0. \end{eqnarray*}

If we plot lg $({\bar {T}}_{i})_{\alpha }$ as the *X* axis and lg ${\bar {E}}_{i}$ as the *Y* axis, the relation between the energy density and the averaged period can be expressed by a straight line whose slope is −1. The IMF component of the white noise series should be distributed on the line in theory; however, the actual application produces little deviation, and the confidence interval for the energy spectrum distribution of white noise is thus presented as follows:

(9) \begin{eqnarray*} \lg \bar {{E}_{i}}=-\lg {\left\{\bar {{T}_{i}}\right\}}_{\alpha }\pm \alpha \sqrt{2/N}{e}^{\lg \left[{\left(\bar {{T}_{i}}\right)}_{\alpha /2}\right]}. \end{eqnarray*}

In the formula, *α* is the significance level.

## Results and Discussion

### Linear change trend

**Figure 2:**
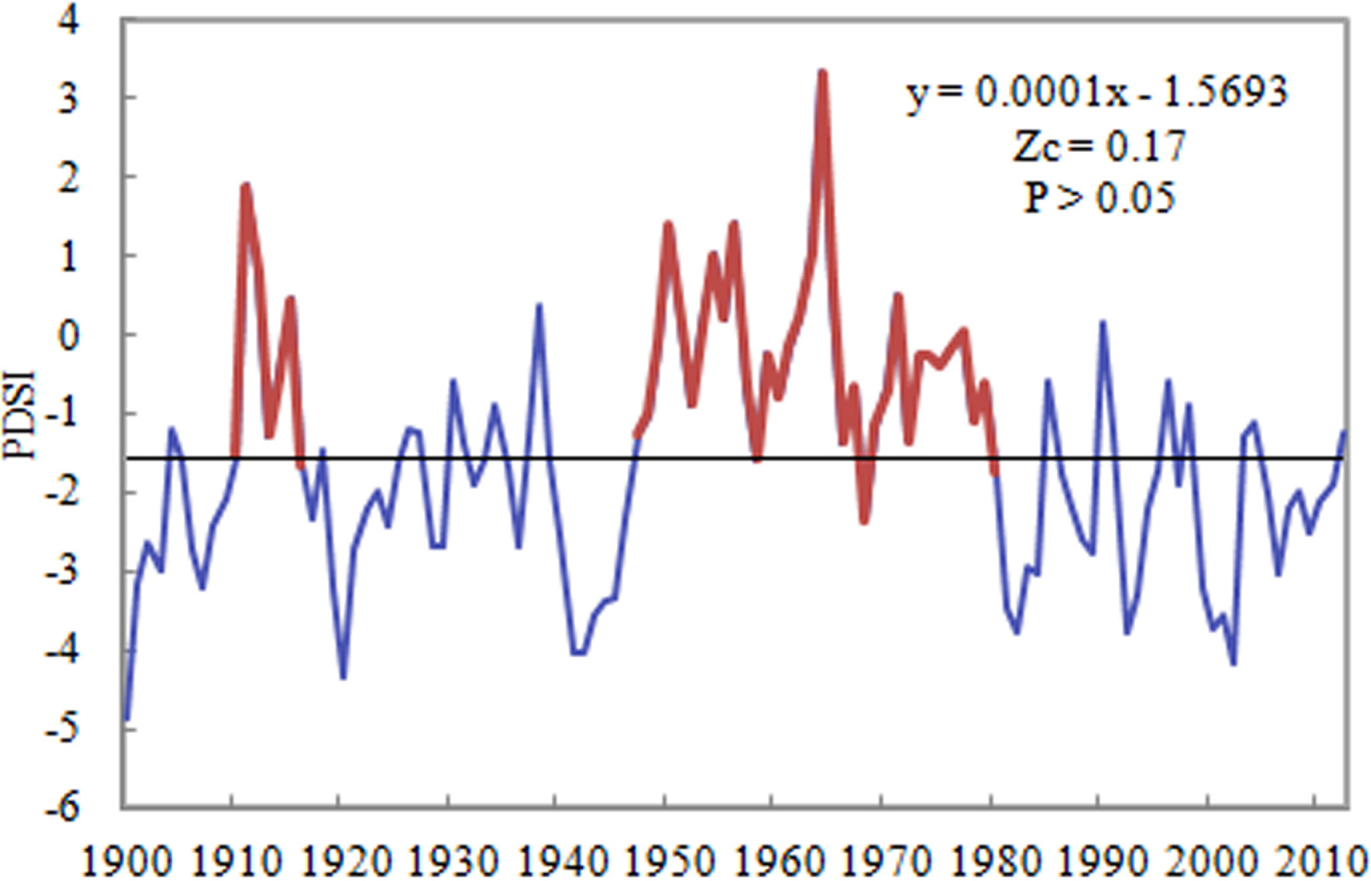
The linear trend of PDSI in the Shandong province of China during 1900–2012.

The Mann–Kendall (MK) statistical test revealed a slightly increasing trend in the PDSI in the Shandong Province of East China for 1900–2012, with a linear tendency of 0.001/decade but no statistical significance. A turning point appeared in the late 1950s. PDSI before the 1950s showed a significant upward trend, with a linear rate of 0.28/decade, while PDSI after that decade had a significant downward trend at a linear rate of −0.48/decade. With respect to time period, the PDSI was higher, i.e., at the normal to slightly wet level, during the 1910s and 1950s–1970s and showed gradual decrease during the 1920s–1940s and after the 1980s, suggesting that the PDSI in Shandong has experienced a “wet-dry-wet-dry” process. In particular, the PDSI for 1980–2010 was lower, i.e., at the severe drought level, indicating that the climate has become dry in recent years. Moreover, from 1960 to 1990, the drying linear rate was −0.94/decade in Shandong, slightly lower than that in northern China (−1.07/decade) for the same period (Qian & Zhou, 2014). At the same time, Fig. 2 also shows that the PDSI change is not linear and shows a strong nonlinear and unstable variation trend. Therefore, we should use a nonlinear method to analyze the nonlinear change trend of the Shandong PDSI.

### Nonlinear change trend

**Figure 3:**
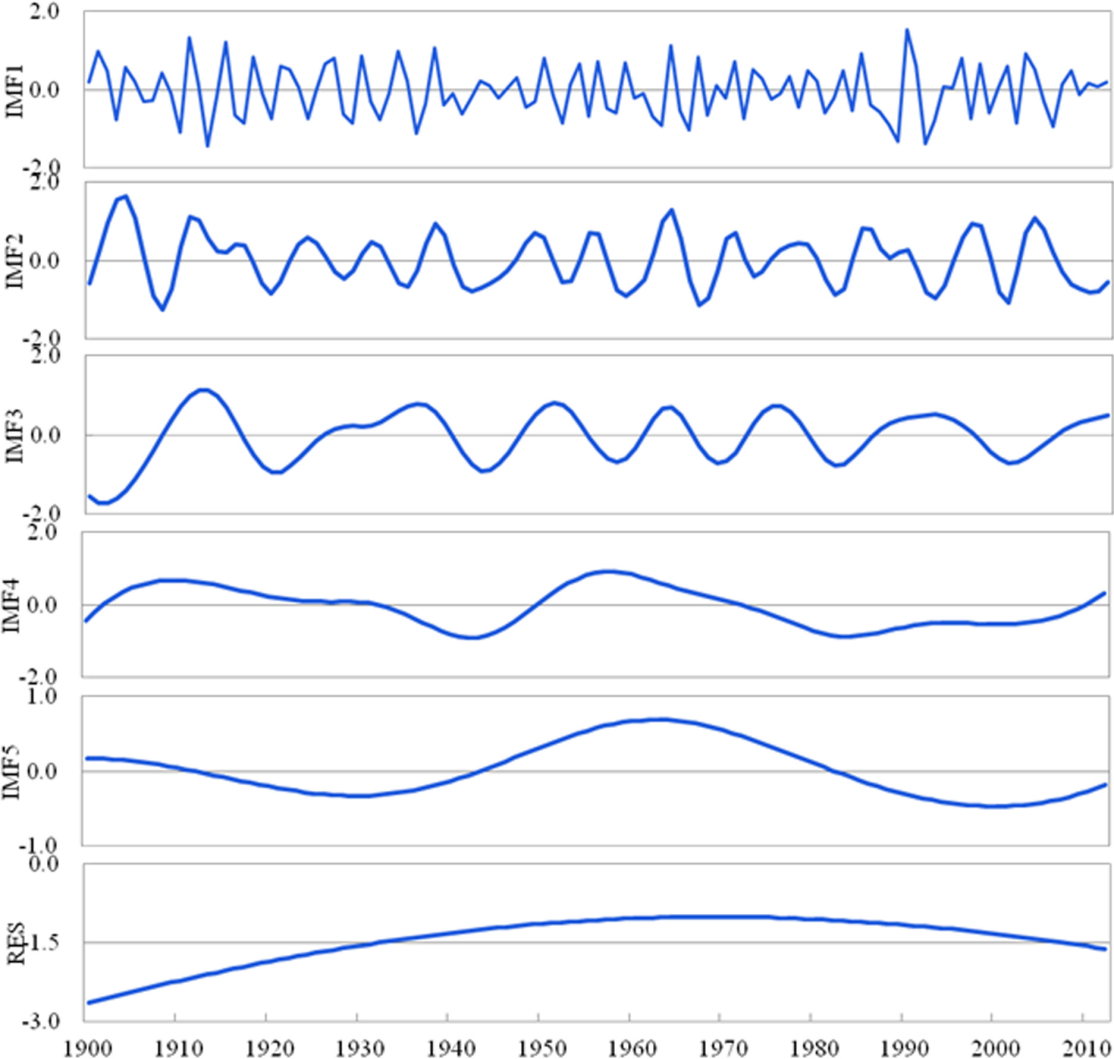
Decomposition of the PDSI at the inter-annual (IMF1, IMF2), inter-decadal (IMF3) and multi-decadal (IMF4, IMF5, RES) scales using the EEMD method from 1900 to 2012 in Shandong.

**Figure 4:**
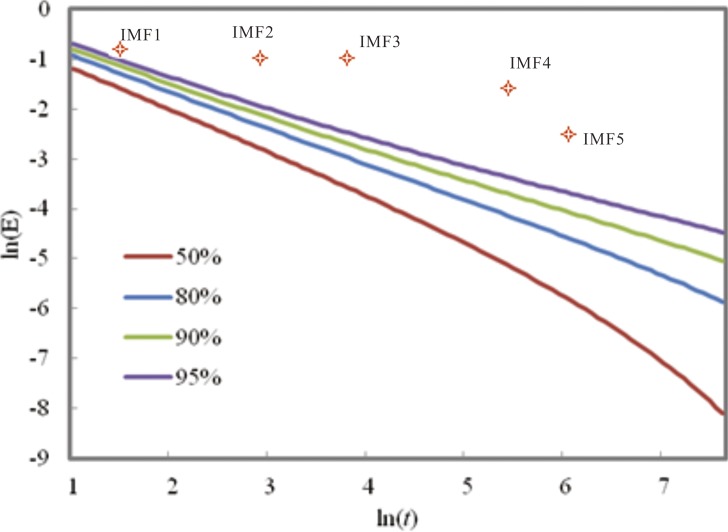
Significance test for the IMF of the PDSI from 1900 to 2012 in Shandong.

The EEMD decomposition results show that the PDSI had five IMF components (IMF1-5) and one trend component (RES) in Shandong for 1900–2012. The fluctuation characteristics from high frequency to low frequency at different time scales can be reflected by each IMF component, determined by a significance test. Concurrently, Fig. 4 indicates that each IMF component has certain inherent scale characteristics (cycle) and also shows the significance test for the IMF1-5 of the PDSI from 1900 to 2012 in Shandong. The IMF1-5 is above the 95% confidence interval, indicating that all the IMFs are significant components and include much information with actual physical meaning. As shown in Figs. 3 and 4, the PDSI changes from 1900 to 2012 show relatively stable quasi periodicity; the PDSI for 1900–2012 has quasi-3-year (IMF1) and quasi-7-year (IMF2) climate variability at the inter-annual scale and quasi-14-year (IMF3), quasi-46-year (IMF4) and quasi-65-year (IMF5) climate variability at the decadal scale. These IMF components contain the periodic changes of climatic systems under external force, as well as nonlinear feedback. Compared to linear methods, such as the MK trend test, EEMD can be an excellent method to distinguish the scale cycles and nonlinear trend of non-linear and non-stationary signals.

The variance contribution rate illustrates the effect of the signal fluctuation frequency and amplitude at each scale on the general characteristics of the available raw data. The variance contribution rate of each component for the PDSI change is stated in Table 2. The contribution of IMF1 towards PDSI variance of the quasi-3-year is the greatest, reaching 19.5% (Table 2), indicating that the PDSI variation is more unstable at this time scale (Fig. 3). The amplitude of the PDSI strongly oscillates with an increase–decrease–increase trend and is significantly higher during the 1910s, 1930s, 1960s, the late 1980s and early 1990s than during other time periods. IMF2 contributes to approximately 19.2% of the PDSI variance of the quasi-7-year cycle, indicating higher PDSI in the mid-1900s, early 1910s and mid-1960s. IMF3 contributes to 18.9% of the quasi-14-year PDSI change, indicating a relatively larger amplitude from the 1900s to 1910s. IMF4 contributes to 13.1% of the PDSI variance of the quasi-46-year cycle, indicating that the PDSI amplitude increases at this time scale. However, IMF5 contributes to 5.9% of the PDSI variance of the quasi-65-year cycle, indicating that the PDSI amplitude is basically stable and has slight instability of variation at this time scale. The trend components contribute to 23.3% of the variance, indicating that the PDSI in Shandong for 1900–2012 has a nonlinear change, increases before the late 1950s and decreases after the late 1950s.

**Table 2:** Contribution rates of EEMD decomposition for PDSI for 1900–2012 in Shandong.

IMF components	IMF1	IMF2	IMF3	IMF4	IMF5	RES
Period (year)	3	7	14	46	65	–
Contribution (%)	19.5%	19.2%	18.9%	13.1%	5.9%	23.3%

### Correlation between PDSI and PDO, ENSO, SOI, SHI

Table 3 clearly shows the periods of PDSI, PDO, SHI, SOI and ENSO at different scales. It is shown that the period of PDSI is basically consistent with those of the circulation factors. Therefore, it is reasonable to analyze the relationships between PDSI and the different circulations (PDO, ENSO, SO and SHI) at different scales.

**Table 3:** The periods (year) of PDSI, PDO, SHI, SOI and ENSO at different scales.

Factor	IMF1	IMF2	IMF3	IMF4	IMF5
PDSI	3.1	7.1	14.1	45.7	64.6
PDO	2.8	5.8	15.9	44.9	63.5
ENSO	3.5	6.2	15.9	30.7	63.0
SHI	2.8	5.8	10.1	27.1	90.9
SOI	3.2	6.2	11.1	23.2	47.6

The Pearson correlation coefficients between the decomposition for PDSI and the decompositions of ENSO, SHI, and SOI for 1900–2010 at different time scales have been calculated in this study and shown in Table 4. It can be observed that the original PDSI (ORP) and the IMF4 and IMF5 of the decomposed PDSI at the annual scale have significant (*P* < 0.01) negative correlation with the PDO and ENSO; only IMF5 has significant (*P* < 0.01) negative correlation with the SOI and SHI. In spring, the ORP and IMF4 and IMF5 of the decomposed PDSI show significant (*P* < 0.001) correlation only with PDO; some IMF4 and IMF5 of the decomposed PDSI show significant (*P* < 0.01) correlation only with SHI and SOI. In summer, the original PDSI (ORP) and the IMF4 and IMF5 of the decomposed PDSI show significant (*P* < 0.01) correlation with PDO, SOI and SHI; IMF1-5 also shows significant (*P* < 0.05) correlation with ENSO. In autumn, the ORP and the IMF4 and IMF5 of the decomposed PDSI show significant (*P* < 0.01) correlation with PDO, ENSO and SOI; IMF1 also shows significant (*P* < 0.05) correlation with ENSO and SOI. In winter, the ORP and the IMF3 and IMF5 of the decomposed PDSI exhibit significant (*P* < 0.01) correlation with PDO and ENSO; the correlation between IMF3 and SHI and the correlation between IMF4 and PDO are also significant (*P* < 0.01). In general, it can be observed from Table 4 that ORP at different scales is mainly influenced by PDO and ENSO of the same year, and the IMF4 and IMF5 at some scales show significant correlation with the four atmospheric circulations (PDO, SHI, SOI and ENSO), which indicates that the effect of atmospheric circulation on PDSI at the multi-decadal variability scale is more important than at the annual (ORP), inter-annual (IMF1, IMF2) and inter-decadal (IMF3) scales. For example, although ORP, IMF1, and IMF2 of PDSI show no obvious correlation with SOI and SHI during different seasons, the correlations between the IMF4 and IMF5 and the SOI and SHI are significant.

**Table 4:** The Pearson correlation coefficient between decomposition for PDSI and the decomposition of ENSO, SHI, and SOI for 1900–2010 at different time scales (yellow, green and red mean *P* < 0.001, 0.01, and 0.05 significance levels, respectively).

		PDSI					
Factor	Scale	ORP	IMF1	IMF2	IMF3	IMF4	IMF5
PDO	Year	−0.386^a^	−0.158	0.033	−0.095	−0.748^a^	−0.931^a^
	Spring	−0.376^a^	−0.162	−0.029	−0.152	−0.625^a^	−0.676^a^
	Summer	−0.262^b^	−0.118	−0.025	−0.049	−0.284^b^	−0.936^a^
	Autumn	−0.287^b^	−0.199	−0.191	−0.235^c^	−0.259^b^	−0.610^a^
	Winter	−0.412^a^	−0.169	0.029	−0.418^a^	−0.623^a^	−0.806^a^
ENSO	Year	−0.276^b^	−0.188	0.139	0.024	−0.477^a^	−0.279^b^
	Spring	−0.142	−0.101	0.210	0.163	0.022	−0.091
	Summer	−0.175	−0.215^c^	0.245^c^	−0.290^b^	−0.247^c^	−0.334^a^
	Autumn	−0.364^a^	−0.309^b^	−0.108	−0.001	−0.463^a^	−0.226^c^
	Winter	−0.276^b^	−0.236^c^	0.209^c^	−0.339^a^	−0.172	−0.933^a^
SOI	Year	0.150	0.067	−0.023	0.209	0.129	0.684^a^
	Spring	0.049	0.001	−0.075	0.032	0.291	0.502^a^
	Summer	0.121	0.141	0.039	−0.155	0.401^a^	0.865^a^
	Autumn	0.259^b^	0.257^b^	0.173	0.024	0.401^a^	0.630^a^
	Winter	0.104	0.104	−0.058	0.155	0.111	−0.340^a^
SHI	Year	−0.109	−0.121	−0.030	0.120	0.023	0.294^b^
	Spring	0.050	−0.101	−0.048	0.192	0.432^a^	0.265^b^
	Summer	−0.206^c^	−0.129	0.074	0.374^a^	0.298^b^	−0.735^a^
	Autumn	−0.041	0.025	−0.214^c^	0.302^b^	0.094	−0.574^a^
	Winter	−0.017	0.066	−0.075	−0.338^a^	−0.041	0.569^a^

**Notes.**

a Yellow.

b Green.

c Red.

**Figure 5:**
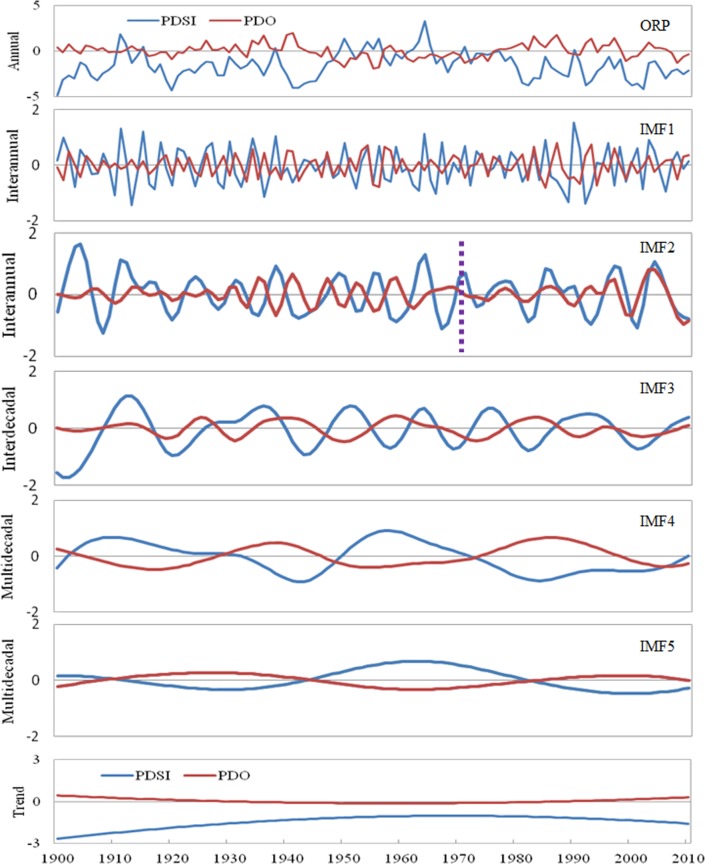
The changes of PDSI and PDO at the original annual scale and decompositions of PDSI and PDO at the inter-annual (IMF1, IMF2), inter-decadal (IMF3), and multi-decadal (IMF4, IMF5, Trend) scales using the EEMD method during 1900–2010.

**Figure 6:**
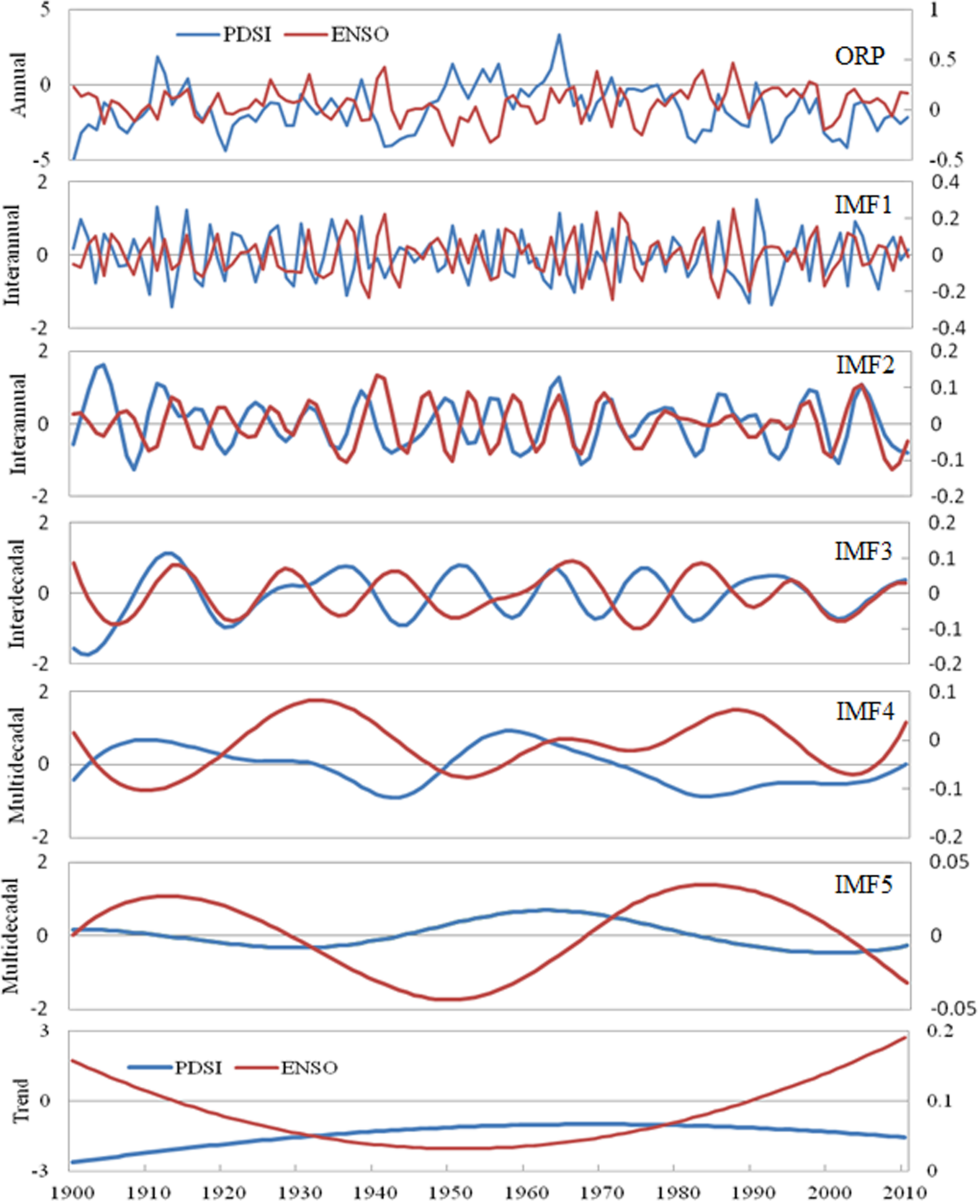
The changes of PDSI and ENSO at the original annual scale and decompositions of PDSI and ENSO at the inter-annual (IMF1, IMF2), inter-decadal (IMF3), and multi-decadal (IMF4, IMF5, Trend) scales using the EEMD method during 1900–2010.

It can be shown from Table 4 that the effect of PDO and ENSO on PDSI is relatively more important than that of other factors. Thus, this study takes the relationship between the PDSI and the PDO and ENSO as an example to analyze the impact of atmospheric circulation on PDSI at different scales (Figs. 5 and 6). For 1900–2010, the Pearson correlation coefficient between the annual PDSI and PDO is −0.386, which is obviously lower than that of IMF4 (−0.784) and IMF5 (−0.931) at the multi-decadal variability scale. Concurrently, Fig. 5 shows that the correlation between the IMF1, IMF2, and IMF3 of the decomposed PDSI and the PDO is very weak; however, the changes of PDSI and PDO at the multi-decadal scale (IMF4 and IMF5) have strong synchronization. The above results show that the PDSI has negative correlation with the PDO at different scales. As shown in Fig. 5, before 1970, the correlation coefficient between PDSI and PDO at the IMF2 scale is −0.365, which is significant at the 0.01 level; however, after 1970, their correlation becomes positive and the coefficient is 0.582, which is significant at the 0.001 level. It illustrated that PDO has a weak effect on the wet and dry variation in Shandong at the IMF2 scale after 1970, while other factors possibly have a strong influence on it. Thus, we can state that the effect of atmospheric circulation on PDSI is unstable at the inter-annual scale. Similarly, Fig. 6 also indicates that the correlations between PDSI and ENSO at the IMF2 and IMF3 scales transfer between positive and negative correlation at different times, and the correlations between PDSI and ENSO at the IMF1, IMF2, and IMF3 scales are lower than those at the IMF4 and IMF5 scales.

How can the PDO and ENSO cause the drought change in the Shandong province? Here, to easily understand the influence mechanism of PDO and ENSO on drought, we can simply describe it as follows. Actually, PDO and ENSO can lead to global exceptional precipitation in some areas, exerting influence on the change of wet and dry (Wang et al., 2014). During the cold phases of PDO and ENSO, dry air over the Pacific Ocean is transmitted to the study area in the form of upper-air wind, causing drought to occur. When PDO and ENSO are in the warm phase, humid air will be transferred into the study area, and as a result, the precipitation becomes higher than average for the Shandong province. Additionally, we can see in Figs. 5 and 6 that the influence of PDO and ENSO on drought in the study area is unstable and that these two factors interact with each other to some degree. When PDO and ENSO are in-phase, the change from dry to wet obviously strengthens. When PDO is in the warm phase, an El Niño event is more likely to occur and drought is more serious. When PDO is in the cold phase, La Niña events occur more frequently, and precipitation increases significantly in the study area, thereby obviously reducing the drought intensity. The result will have important instruction significance for drought risk prediction in the study area.

## Conclusions

Based on the PDSI time series in Shandong from 1900 to 2012, the multi-scale linear and nonlinear characteristics of PDSI variability were analyzed using linear regression, the Mann–Kendall test and the EEMD method, and its relations to PDO, ENSO, SO and SH were detected using the Pearson correlation analysis method. The primary findings include the following:

A long-term transformation from a significant wetting to a significant drying trend is tested with respect to PDSI for Shandong near the late 1950s. Although the PDSI shows no statistically significant linear change trend for 1900–2012, before (after) the late 1950s, the PDSI shows a significant upward (downward) trend (*P* < 0.01), with a linear rate of 0.28/decade (−0.48/decade).

The PDSI change shows not only a linear trend but also a strong nonlinear variation trend, and its changes are clearly exhibited at the inter-annual scale (quasi-3 and quasi-7-year), inter-decadal scale (quasi-14-year) and multi-decadal scale (quasi-46 and quasi-65-year). All five quasi-periodic components are above the 95% confidence interval, indicating that they contain significant information with actual physical meaning. The variance contribution rate of IMF1 is the largest, reaching 19.5%; the contribution of IMF2 is also large, reaching 19.2%; the variance contribution rates of IMF3 and IMF4 are relatively small, with values of 18.9% and 13.1%, respectively; the contribution of IMF5 is the smallest, with only a value of 5.9%. These results indicate that the inter-annual change had a huge impact on the overall PDSI change in Shandong, East China.

The PDSI in Shandong exhibits negative correlation with PDO and ENSO, and their relationship at the multi-decadal scale is more significant than at the inter-annual scale and inter-decadal scale. The PDSI shows positive correlation with SOI, and their relationship is significant only at the multi-decadal scale, e.g., IMF5. The PDSI has positive or negative correlation with SHI at different scales, and their relationship at the multi-decadal scale is more significant than at the inter-annual scale. This indicates that the effect of atmospheric circulation on PDSI at the multi-decadal variability scale is more important than at the inter-annual and inter-decadal scale. Thus, a model aiming to predict the drought of East China or the East Asia region should take into account the variation of atmospheric circulation (such as PDO, ENSO, SO, SH, etc.) at the multi-decadal scale, which should be a major item on future research agendas.

A secular transition from significant negative to significant positive correlation is detected in the IMF2 of PDSI and PDO near the 1970s. For 1900–1969, the correlation coefficients between PDSI and PDO at the IMF2 scale is −0.365 (*P* < 0.01); however, their correlation coefficient is 0.582 for 1970–2010 (*P* < 0.001), which implies that the effect of PDO on PDSI is unstable at the inter-annual scale. Accordingly, the mechanism linking PDSI and atmospheric circulation at the inter-annual scale needs further study in the future.

We employed the EEMD method to separate inter-annual and inter-decadal variation trends from observation sequences for several years and to separate the overall trend of climate change from the time series of climatological observations for several years, which will be helpful to probe the issues of global or regional climate change. Although the impact of human actives on climate is important, climate change is primarily controlled by internal variations of the climatic system at the inter-annual and inter-decadal scales, which exhibit significant natural variability. These study results show that the climate variation at the inter-annual scale is more unstable and that the inter-decadal and multi-decadal scales are affected by more natural factors. Thus, more comprehensive and in-depth analysis is needed in future study.

## Supplemental Information

10.7717/peerj.1289/supp-1 Supplemental Information 1 The data of PDSI-PDO-SOI-ENSO-SHI
