# Supplementary material for: The nonlinear variation of drought and its relation to atmospheric circulation in Shandong Province, East China
Source: PeerJ. 2015 Oct 27;3:e1289. doi: 10.7717/peerj.1289 (PMC4627919; doi:10.7717/peerj.1289)
Supplement: Supplemental Information 1 [file peerj-03-1289-s001.zip › data.docx]

| Yerar | PDSI | PDO | SHI | ENSO | SOI |
| --- | --- | --- | --- | --- | --- |
| 1900 | -4.874688 | 0.454167 | 0.535933 | 0.2333333 | -2.291667 |
| 1901 | -3.150417 | -0.12917 | 0.634512 | 0.1366667 | -0.041667 |
| 1902 | -2.641042 | 0.7675 | 0.54821 | 0.165 | 0.1666667 |
| 1903 | -2.996354 | 0.163333 | 0.817586 | 0.1316667 | 1.1416667 |
| 1904 | -1.196146 | -0.2525 | 0.476911 | -0.134167 | 1.1333333 |
| 1905 | -1.581458 | 0.641667 | 0.521514 | 0.0966667 | -6.175 |
| 1906 | -2.732917 | 0.485833 | 0.25041 | 0.0675 | 0.6333333 |
| 1907 | -3.191875 | 0.118333 | 0.801533 | -0.015833 | -0.566667 |
| 1908 | -2.431979 | 0.378333 | -0.02584 | -0.1075 | 0.85 |
| 1909 | -2.056771 | -0.11667 | 0.730104 | -0.005833 | 0.6416667 |
| 1910 | -1.525625 | -0.07583 | 0.503783 | 0.0533333 | 3.675 |
| 1911 | 1.8946875 | -0.15667 | 0.121808 | -0.086667 | -1.816667 |
| 1912 | 0.8027083 | 0.1525 | 0.749995 | 0.1891667 | -3 |
| 1913 | -1.276042 | 0.574167 | 0.03047 | 0.1116667 | -2.125 |
| 1914 | -0.516146 | 0.16 | -0.7118 | 0.1366667 | -2.083333 |
| 1915 | 0.4573958 | 0.1775 | -0.1532 | 0.215 | 0.0083333 |
| 1916 | -1.645625 | -0.50917 | 0.353905 | -0.059167 | 1.7166667 |
| 1917 | -2.318958 | -0.425 | -0.14229 | -0.12 | 6.075 |
| 1918 | -1.457188 | -0.13583 | -0.09929 | 0.0083333 | 0.2166667 |
| 1919 | -3.215521 | -0.10083 | -0.25034 | 0.1675 | -3.375 |
| 1920 | -4.34 | -0.90667 | 0.400308 | -0.033333 | 0.1333333 |
| 1921 | -2.710833 | -0.0975 | -0.01645 | -0.041667 | 1.9416667 |
| 1922 | -2.184167 | -0.19667 | -0.46235 | 0.0033333 | 1.1083333 |
| 1923 | -1.973229 | 0.4825 | -0.04597 | 0.0191667 | -1.075 |
| 1924 | -2.426042 | 0.136667 | -0.04821 | 0.1075 | 1.0916667 |
| 1925 | -1.628229 | 0.190833 | 0.041392 | 0.0391667 | -0.708333 |
| 1926 | -1.180104 | 1.16 | 0.031129 | 0.3075 | -1.508333 |
| 1927 | -1.240938 | 0.140833 | 0.339263 | 0.1491667 | 0.75 |
| 1928 | -2.671771 | 0.155833 | 0.336897 | 0.0925 | 1.4583333 |
| 1929 | -2.701667 | 0.400833 | 0.555192 | 0.0716667 | 1.6583333 |
| 1930 | -0.598542 | -0.10417 | 0.241082 | 0.0925 | 0.175 |
| 1931 | -1.354375 | 0.738333 | 0.402033 | 0.3516667 | 0.7583333 |
| 1932 | -1.910104 | -0.02083 | 0.258968 | 0.065 | -1.875 |
| 1933 | -1.583438 | -0.68 | 0.000963 | -0.039167 | 0.15 |
| 1934 | -0.908125 | 1.1825 | 0.703562 | -0.0925 | -0.158333 |
| 1935 | -1.671042 | 0.798333 | 0.374348 | 0.025 | 0.4833333 |
| 1936 | -2.659896 | 1.730833 | 0.389622 | 0.1133333 | -0.533333 |
| 1937 | -1.270417 | 0.324167 | 0.554151 | 0.0916667 | 0.0666667 |
| 1938 | 0.3816667 | 0.155 | 0.290303 | -0.100833 | 2.5 |
| 1939 | -1.610208 | 0.065 | 0.105538 | -0.094167 | 1.48E-16 |
| 1940 | -2.571563 | 1.769167 | 0.432919 | 0.31 | -4.108333 |
| 1941 | -4.0525 | 1.994167 | -0.43305 | 0.43 | -4.225 |
| 1942 | -4.012813 | 0.465833 | -0.09378 | 0.0033333 | -0.058333 |
| 1943 | -3.563854 | 0.114167 | 0.255572 | -0.185833 | 1.0916667 |
| 1944 | -3.381667 | -0.12667 | 0.372795 | -0.005833 | -0.75 |
| 1945 | -3.320625 | -0.19 | -0.19965 | 0.0175 | 1.3416667 |
| 1946 | -2.338646 | -0.58333 | -0.54978 | 0.0108333 | -2.216667 |
| 1947 | -1.260104 | 0.5 | 0.39629 | 0.0641667 | 0.4916667 |
| 1948 | -1.004167 | -0.87417 | -0.31271 | -0.0025 | -0.85 |
| 1949 | -0.069271 | -1.22833 | 0.168145 | -0.190833 | -0.575 |
| 1950 | 1.3910417 | -1.81 | -0.35346 | -0.349167 | 4.3 |
| 1951 | 0.1338542 | -0.76917 | 0.318718 | -0.071667 | -1.875 |
| 1952 | -0.878854 | -0.86583 | 0.632413 | -0.1075 | -0.916667 |
| 1953 | 0.1032292 | -0.15667 | 0.192001 | 0.0383333 | -2.233333 |
| 1954 | 1.0204167 | -0.29083 | 0.527913 | -0.166667 | 0.675 |
| 1955 | 0.19875 | -1.94833 | -0.15201 | -0.316667 | 2.7083333 |
| 1956 | 1.4030208 | -1.80417 | 0.0112 | -0.2525 | 2.875 |
| 1957 | -0.653438 | 0.2275 | 0.358802 | 0.105 | -1.341667 |
| 1958 | -1.570938 | 0.643333 | 0.308377 | 0.145 | -1.508333 |
| 1959 | -0.259479 | -0.02667 | -0.38856 | 0.0425 | -0.358333 |
| 1960 | -0.782708 | 0.0575 | 0.080378 | 0.0283333 | 0.9416667 |
| 1961 | -0.149375 | -0.8175 | -0.62447 | -0.129167 | -0.108333 |
| 1962 | 0.2310417 | -1.15833 | 0.241624 | -0.090833 | 1.0583333 |
| 1963 | 1.0434375 | -0.68583 | -0.26807 | 0.2158333 | -0.916667 |
| 1964 | 3.3283333 | -0.77 | 0.005825 | 0.0725 | 1.3083333 |
| 1965 | 0.6051042 | -0.31417 | -0.01549 | 0.1983333 | -2.541667 |
| 1966 | -1.372396 | -0.45917 | 0.258247 | 0.2275 | -1.583333 |
| 1967 | -0.66 | -0.73417 | 0.230303 | -0.1325 | 0.9083333 |
| 1968 | -2.344271 | -0.40333 | 0.164914 | 0.0683333 | 0.525 |
| 1969 | -1.137396 | -0.09833 | 0.472194 | 0.3825 | -2.066667 |
| 1970 | -0.678229 | -0.3975 | 0.116672 | 0.0875 | 0.75 |
| 1971 | 0.50125 | -1.29083 | -0.22806 | -0.165 | 3.025 |
| 1972 | -1.344896 | -0.92167 | 0.082637 | 0.225 | -2.491667 |
| 1973 | -0.247917 | -0.80417 | -0.16289 | 0.1 | 1.7666667 |
| 1974 | -0.277083 | -0.33667 | 0.340537 | -0.183333 | 3 |
| 1975 | -0.388542 | -1.10167 | -0.65558 | -0.25 | 3.8166667 |
| 1976 | -0.148854 | 0.008333 | 0.168312 | -0.000833 | 0.25 |
| 1977 | 0.0330208 | 0.230833 | 0.129068 | 0.1058333 | -3.083333 |
| 1978 | -1.078229 | 0.235833 | -0.5658 | 0.0408333 | -1.1 |
| 1979 | -0.623125 | 0.335 | 0.204862 | 0.1333333 | -0.333333 |
| 1980 | -1.745938 | 0.6025 | 0.423541 | 0.2033333 | -1.125 |
| 1981 | -3.477708 | 0.918333 | 0.549839 | 0.1216667 | 0.0083333 |
| 1982 | -3.776458 | 0.114167 | -0.04575 | 0.3058333 | -3.883333 |
| 1983 | -2.957813 | 1.648333 | -0.31141 | 0.395 | -3.308333 |
| 1984 | -3.039479 | 0.8375 | 0.121347 | 0.1166667 | -0.375 |
| 1985 | -0.602813 | 0.449167 | -0.00149 | 0.005 | 0.0916667 |
| 1986 | -1.766146 | 1.239167 | 0.117491 | 0.1733333 | -1.191667 |
| 1987 | -2.216458 | 1.820833 | 0.184707 | 0.4741667 | -3.991667 |
| 1988 | -2.597292 | 0.531667 | 0.065628 | 0.1866667 | 2.1833333 |
| 1989 | -2.788333 | -0.17917 | 0.167071 | -0.076667 | 1.7166667 |
| 1990 | 0.1577083 | -0.35583 | -0.58423 | 0.0758333 | -1.15 |
| 1991 | -1.234167 | -0.41917 | 0.12469 | 0.1791667 | -2.775 |
| 1992 | -3.764896 | 0.928333 | 0.21679 | 0.2158333 | -3.683333 |
| 1993 | -3.312708 | 1.416667 | 0.252954 | 0.2216667 | -3 |
| 1994 | -2.185938 | -0.15167 | -0.27773 | 0.1333333 | -3.658333 |
| 1995 | -1.722396 | 0.6425 | -0.05065 | 0.2075 | -0.841667 |
| 1996 | -0.604896 | 0.640833 | 0.605018 | 0.1291667 | 1.55 |
| 1997 | -1.893125 | 1.460833 | -0.24993 | 0.28 | -3.15 |
| 1998 | -0.910313 | 0.245833 | -0.14747 | 0.2525 | -0.908333 |
| 1999 | -3.186042 | -1.06333 | -0.21347 | -0.1975 | 2.2916667 |
| 2000 | -3.742396 | -0.59 | -0.26001 | -0.149167 | 2.1916667 |
| 2001 | -3.567708 | -0.5625 | -0.35676 | -0.058333 | 0.0166667 |
| 2002 | -4.162188 | 0.220833 | -0.5506 | 0.16 | -1.816667 |
| 2003 | -1.302917 | 0.969167 | 0.23756 | 0.2066667 | -1.1 |
| 2004 | -1.116042 | 0.345 | -0.14125 | 0.0833333 | -1.641667 |
| 2005 | -2.006146 | 0.375 | 0.879201 | 0.0758333 | -1.425 |
| 2006 | -3.03 | 0.190833 | 0.488359 | 0.1175 | -0.625 |
| 2007 | -2.213646 | -0.19583 | 0.167445 | 0.0683333 | 0.1166667 |
| 2008 | -1.971667 | -1.2925 | 0.189168 | -0.0575 | 3.4 |
| 2009 | -2.5175 | -0.6125 | 0.541048 | 0.1825 | -0.233333 |
| 2010 | -2.107708 | -0.3125 | 1.144442 | 0.165 | 2.4916667 |
| 2011 | -1.898542 | -1.23083 |  |  | 4.3083333 |
| 2012 | -1.257188 | -1.1 |  |  | -0.291667 |
